# Improving Documentation of Intravenous Cannulation in a Resource-Limited Teaching Hospital: A Two-Cycle Quality Improvement Audit

**DOI:** 10.7759/cureus.96186

**Published:** 2025-11-06

**Authors:** Wael Ahmedalbashier Mohammed Ahmed, Rouida Elfadil Mohamed Ahmed Aboagla, Mohammed Osman Ahmed Osman, Sara Isam Eldin Ibrahim Elshafie, Eman Abubakeralsideeg Diaaldeen Mohamed, Marwa Mohammed Khairy Fadel, Aya Eltom Hajo Elsheikh, Asim Eisa, Elkhatab Abdulrahman Mustafa Abdulrahman, Fatima Ahmed Mohamed Mustafa, Weaam Ibrahim Bukhari, Sara Abdalla Osman Mohamed, Mohey Aldien Ahmed Elamin Elnour, Mohamed Saifeldin Sharif Hussein, Lina Ahmed, Manal Hashim Saeid Othman, Mohanad Elsafi Mossaad Elbashier, Khadega Osman Abbas Alhudi, Nmarig Elawad Ahmed Ali, Elwathig Abdalla

**Affiliations:** 1 Emergency, Al-Managil Teaching Hospital, Al-Managil, SDN; 2 Pediatric Surgery, Abu El-Reesh Children’s Hospital, Cairo University, Cairo, EGY; 3 Internal Medicine, Al-Managil Teaching Hospital, Al-Managil, SDN; 4 Emergency, Atbara Teaching Hospital, Atbara, SDN; 5 General Surgery, Al-Managil Teaching Hospital, Al-Managil, SDN; 6 Obstetrics and Gynecology, Al-Managil Teaching Hospital, Al-Managil, SDN; 7 Trauma and Orthopedics, Al-Managil Teaching Hospital, Al-Managil, SDN; 8 Orthopedic Surgery, Sudan Medical Specialization Board, Khartoum, SDN

**Keywords:** clinical audit, documentation, intravenous cannulation, patient safety, peripheral intravenous catheter, quality improvement, resource-limited settings

## Abstract

Background: Incomplete documentation of intravenous (IV) cannulation is a common problem that compromises patient safety, accountability, and continuity of care. This study aimed to evaluate the impact of introducing a standardized IV cannula documentation sticker on compliance with documentation standards in a resource-limited hospital.

Methods: A two-cycle quality improvement (QI) audit was conducted at Al-Managil Teaching Hospital, Sudan, in 2025. In Cycle 1 (March 20-April 2), 50 inpatient records were retrospectively reviewed for compliance with IV cannula documentation standards. A one-month intervention was then implemented, introducing a standardized documentation sticker supported by staff training, posters, and ward-level reminders. Cycle 2 (June-September) reviewed another 50 records prospectively. Data were analyzed using frequencies, percentages, and chi-square tests, with p < 0.05 considered statistically significant.

Results: Documentation compliance improved markedly across all parameters following the intervention. In Cycle 1, compliance ranged from 0 to 68%, while in Cycle 2, most parameters reached ≥ 95%. For example, documentation of insertion site improved from 4% to 100%, aseptic technique from 2% to 96%, and complications from 4% to 96%. Flushes/patency checks, while significantly improved (0% to 80%), remained lower than other domains. All improvements were statistically significant (p < 0.001).

Conclusions: In a resource-limited setting, a structured documentation sticker, combined with staff education, achieved near-universal compliance with IV cannula documentation standards. This low-cost, scalable intervention has the potential to strengthen patient safety, accountability, and quality of care. Sustaining progress will require re-auditing, ongoing staff reinforcement, and targeted focus on maintenance documentation.

## Introduction

Intravenous (IV) cannulation is one of the most frequently performed invasive procedures in hospital settings, providing essential access for fluids, medications, nutrition, and blood products. Despite its routine use, IV cannulation is not without risks. Complications such as phlebitis, infiltration, occlusion, and catheter-related bloodstream infections are common, particularly when insertion and maintenance are not performed according to established standards. These complications not only prolong hospital stays but also increase healthcare costs and patient morbidity [1].

Effective documentation of IV cannula insertion and care is a cornerstone of safe clinical practice. Proper records, including details of insertion time, site, cannula size, and subsequent monitoring, enable healthcare teams to detect complications early, facilitate continuity of care, and ensure accountability. International guidelines stress that standardized documentation is essential to maintain device safety, reduce infection risk, and optimize outcomes [2].

However, audits across diverse healthcare settings have repeatedly highlighted deficiencies in IV cannula documentation. Missing or incomplete entries regarding insertion sites, dwell times, or patency checks hinder monitoring and contribute to lapses in infection prevention. Studies have demonstrated that such documentation gaps are associated with increased risk of phlebitis and unplanned cannula replacement, thereby compromising patient safety [3]. In resource-limited hospitals such as Al-Managil Teaching Hospital, these challenges are often amplified by staff shortages, the absence of standardized documentation tools, and limited opportunities for continuous training.

Quality improvement (QI) initiatives have proven effective in addressing these gaps. Structured documentation tools, such as stickers or proformas, combined with staff education and feedback, have been shown to significantly improve compliance with documentation standards. The Plan-Do-Study-Act (PDSA) model, in particular, offers a robust framework for implementing, testing, and refining such interventions in real-world hospital environments [4].

By introducing a standardized IV cannula documentation sticker alongside targeted staff training, we aimed to strengthen compliance with documentation standards, enhance patient safety, and establish sustainable practices for ongoing improvement. The objective of this two-cycle QI audit was to evaluate baseline IV cannula documentation practices, implement a standardized documentation sticker accompanied by staff training, and assess the improvement achieved following the intervention. This manuscript presents the findings from the second audit cycle, comparing outcomes before and after the intervention [5].

## Materials and methods

Study design and setting

This QI project was designed as a prospective, two-cycle clinical audit and conducted at Al-Managil Teaching Hospital, Sudan, in 2025. The hospital is a large tertiary facility providing services across key departments, including medicine, pediatrics, obstetrics and gynecology, and surgery. The project was guided by the PDSA model, enabling structured implementation of change, measurement of outcomes, and reassessment in a second audit cycle. Cycle 1 was conducted over a two-week period in May 2025 to establish baseline documentation performance, while Cycle 2 was carried out in September 2025 following the implementation of targeted interventions. Identical audit tools and assessment criteria were used in both cycles to ensure a robust comparison of results.

Study population and sampling

The study population consisted of adult inpatients (≥ 18 years) who had a documented peripheral IV cannula during the study period. A total of 100 patient clinical records were reviewed in each cycle, selected to reflect proportional representation from the four major hospital departments (medicine, surgery, pediatrics, and obstetrics and gynecology). However, the emergency department accounted for the highest proportion of cases due to its role in providing acute clinical care.

Patient age ranged from 18 to 84 years, with the most common clinical indications for cannulation being IV fluid administration, medication infusion, and blood sampling. Peripheral IV cannulas used in the hospital are routinely intended for short-term access (24-72 hours), depending on clinical need and site condition.

Data were extracted prospectively using a standardized audit checklist by two independent auditors. Although the study was not formally powered for statistical comparison, this sample size was deemed adequate to detect meaningful improvements in documentation practices between cycles.

Records were selected using a simple random sampling technique by assigning serial numbers to all eligible files and selecting cases using a random-number table to minimize selection bias. Notes without documented evidence of peripheral IV cannulation or those involving central venous catheters were excluded. Missing data within selected files were treated as “not documented” to ensure full case inclusion without data loss.

Audit cycles and intervention period

The first audit cycle was conducted from March 20 to April 2, 2025, serving as the baseline measurement of IV cannula documentation practices. A one-month intervention period followed between April and May 2025, during which the new documentation strategy was introduced. The second audit cycle was then completed between June and September 2025 to evaluate the impact of the intervention.

Standards and outcome measures

Documentation practices were evaluated using predefined standards for IV cannulation derived from internationally recognized expert consensus guidelines, including those published in the British Journal of Nursing [6,7]. The standards required that clinical records include patient identifiers (name and hospital number), the date and time of cannula insertion, the insertion site and gauge, and the inserter’s name and designation. Additionally, documentation of daily cannula site assessments for complications (e.g., phlebitis or infiltration), flush or patency checks, and any complications was expected. Removal details, including date, time, and indication, were also assessed.

The primary outcome was overall completeness of IV cannula documentation, defined as the presence of all required parameters in accordance with established standards. Secondary outcomes included identifying which parameters were most frequently omitted in each audit cycle.

Intervention

Between the first and second audit cycles, a package of low-cost, practical interventions was introduced to address the deficiencies identified in IV cannula documentation. The primary measure was the design and implementation of a standardized IV cannula documentation sticker (Appendix 1), which functioned as a checklist integrated directly into the patient’s clinical notes. This tool ensured that essential parameters such as insertion details, daily site assessments, flushes, and removal were consistently recorded.

To support correct and consistent use of the sticker, targeted staff training sessions were delivered to doctors, nurses, and other healthcare providers across the participating departments. The sessions were interactive and concise, emphasizing the importance of accurate documentation, demonstrating proper use of the sticker, and highlighting its role in preventing complications and improving patient safety. Training was conducted during routine departmental meetings to avoid service disruption and reduce resource demands.

Visual reminders in the form of posters were also displayed in clinical areas to reinforce adherence. Senior staff and ward supervisors were encouraged to provide verbal prompts during rounds, reminding junior staff to utilize the sticker. Printing of the stickers was supported by the hospital administration, ensuring that the intervention remained cost-neutral and sustainable.

To embed the practice into the routine workflow, staff were instructed to include the sticker as a mandatory component of every cannulation kit. This ensured documentation became a natural step in the clinical process rather than an additional task. Together, these structured, low-cost measures aimed to strengthen accountability, consistency, and patient safety within the constraints of available resources.

Data collection and analysis

Data were extracted from patient records using a structured proforma developed by the audit team. Each documentation parameter was scored dichotomously as either “present” or “absent.” Missing entries were treated as “not documented” to ensure that all selected cases remained included in the analysis.

Data from both audit cycles were compiled and analyzed quantitatively. Frequencies and percentages were calculated for each parameter to describe overall documentation performance.

Comparisons between Cycle 1 and Cycle 2 were assessed using the chi-square test of independence. For each documentation parameter, the observed frequencies of “present” and “absent” responses were arranged into 2 × 2 contingency tables. Statistical significance was defined as p < 0.05. All statistical analyses were conducted using the IBM SPSS Statistics for Windows, Version 29 (Released 2022; IBM Corp., Armonk, New York, United States).

Ethical considerations

Ethical approval was obtained from the Institutional Review Board of Al-Managil Teaching Hospital (approval number: QIP-8-25-0067). All patient data were anonymized prior to analysis to ensure confidentiality.

## Results

In Cycle 1, documentation compliance was suboptimal across most parameters. The patient’s name was recorded in 34 (68%), and age in 23 (46%). In contrast, key details such as date of birth were recorded in two (4%), insertion site in two (4%), and aseptic technique in only one (2%). Accountability indicators were also poorly captured, with the inserter’s role written in two (4%), the inserter’s signature in two (4%), and the remover’s role in zero (0%).

Following the intervention, substantial improvements were observed in Cycle 2. Patient identifiers, insertion and removal details, and staff accountability elements reached near-complete documentation. For example, the patient’s name was documented in 50 (100%), date of birth in 48 (96%), and aseptic technique in 48 (96%). The inserter’s role and signature, as well as the remover’s details, were documented in 50 (100%). Flush and patency checks, although remaining the lowest-performing item, improved markedly from zero (0%) in Cycle 1 to 40 (80%) in Cycle 2. This improvement reflects better consistency in documenting flush or patency assessments when these procedures were performed, rather than implying universal application to all inserted cannulas. All improvements between the two cycles were statistically significant (p < 0.001). These findings are presented in Table 1.

**Table 1:** Comparison of documentation compliance for intravenous cannula insertion and removal across two audit cycles The table shows frequencies and percentages of documentation parameters recorded in Cycle 1 (baseline, n=50) and Cycle 2 (post-intervention, n=50). Data are presented as absolute counts with percentages in parentheses. Chi-square test values and p-values are included to demonstrate the statistical significance of improvements. All differences were statistically significant (p < 0.001).

Parameter	Cycle 1, n (%)	Cycle 2, n (%)	χ²	p-value
Patient’s name written	34 (68.0)	50 (100.0)	19.05	<0.001
Patient’s gender selected	15 (30.0)	50 (100.0)	53.85	<0.001
Patient’s age recorded	23 (46.0)	50 (100.0)	36.99	<0.001
Date of birth recorded	2 (4.0)	48 (96.0)	84.64	<0.001
Insertion date recorded	7 (14.0)	50 (100.0)	75.44	<0.001
Insertion time recorded	2 (4.0)	50 (100.0)	88.89	<0.001
Cannula gauge selected	13 (26.0)	46 (92.0)	38.17	<0.001
Insertion site written	2 (4.0)	50 (100.0)	88.89	<0.001
Insertion indication documented	7 (14.0)	50 (100.0)	75.44	<0.001
Aseptic technique ticked	1 (2.0)	48 (96.0)	88.02	<0.001
Site monitoring ticked	2 (4.0)	50 (100.0)	88.89	<0.001
Complications documented	2 (4.0)	48 (96.0)	84.64	<0.001
Inserter’s name written	8 (16.0)	50 (100.0)	69.77	<0.001
Inserter’s role written	2 (4.0)	50 (100.0)	88.89	<0.001
Inserter’s signature done	2 (4.0)	50 (100.0)	88.89	<0.001
Flushes/patency checks documented	0 (0.0)	40 (80.0)	55.56	<0.001
Removal date written	2 (4.0)	50 (100.0)	88.89	<0.001
Removal time recorded	2 (4.0)	50 (100.0)	88.89	<0.001
Removal indication selected	0 (0.0)	48 (96.0)	88.02	<0.001
Remover’s name documented	3 (6.0)	50 (100.0)	85.25	<0.001
Remover’s role written	0 (0.0)	50 (100.0)	100.00	<0.001
Remover’s signature done	1 (2.0)	50 (100.0)	94.23	<0.001
Additional notes written	2 (4.0)	49 (98.0)	85.99	<0.001

## Discussion

This QI project demonstrated that the introduction of a structured documentation sticker, supported by staff training, led to dramatic improvements in IV cannula documentation. Across all parameters, compliance rose from very low baseline levels to near-universal adherence in the re-audit cycle. For example, documentation of insertion site, aseptic technique, and removal indication exceeded 95% after the intervention. These results provide strong evidence that structured, low-cost strategies can drive significant system-level improvements in resource-constrained settings.

The deficiencies observed in Cycle 1 are consistent with previous studies, which have consistently reported incomplete and inconsistent documentation of peripheral intravenous cannula (PIVC) insertion and care. Marsh et al. highlighted that inadequate record-keeping contributes directly to device failure and associated complications [1], while Biswas et al. documented widespread omissions in a teaching hospital audit [5]. Similarly, Yagnik et al. found low adherence to institutional documentation standards, leaving patients at risk of phlebitis and infection [8]. These findings, together with our baseline results, confirm that inadequate PIVC documentation remains a global challenge.

The improvements achieved in Cycle 2 mirror evidence from structured QI interventions worldwide. Cho and Kim demonstrated that standardized documentation combined with staff education significantly increased compliance with guidelines [9]. Høvik et al. validated structured monitoring instruments such as the PIVC-mini questionnaire (miniQ) as reliable tools for ensuring quality and preventing omissions [10]. In our study, the sticker acted as a visual prompt integrated into the workflow, which likely minimized reliance on memory and reduced the tendency for incomplete records.

Particularly encouraging was the near-complete recording of aseptic technique, insertion indication, and complications, parameters that had been almost entirely neglected in the baseline cycle. This aligns with the recommendations of the Australian Clinical Care Standard [2] and the consensus standards published by Thompson et al. [6], both of which emphasize comprehensive documentation as central to infection prevention and accountability. Alexandrou et al. also showed in a multinational survey that patient outcomes were strongly influenced by adherence to insertion and maintenance practices [3]. Our results demonstrate that these international standards can be achieved even in low-resource hospitals when practical tools are provided.

Despite overall success, the documentation of flushes and patency checks lagged behind other domains, improving from 0% to 80% but not reaching universal compliance. This shortfall has been noted in other settings, where insertion details are prioritized while maintenance documentation is inconsistently performed. Zingg et al. reported that poor recording of maintenance tasks is a key driver of premature catheter failure [7]. Another study found that limited awareness, staff workload, and resource constraints contributed to suboptimal documentation of cannula care [11]. These findings suggest that targeted education, simplified fields, and continuous reinforcement are necessary to achieve full compliance in this area.

Sustainability of change is another important consideration. Taylor et al. emphasized that the PDSA model supports continuous evaluation and embedding of new practices into daily workflow [4]. In our project, reinforcement strategies such as posters, ward reminders, and senior staff oversight played a role similar to digital feedback systems in wealthier settings. Henderson et al. confirmed that auditing combined with feedback improves both documentation and staff accountability [12]. Our experience suggests that such reinforcement, even when low-tech, is crucial for sustaining improvement in resource-limited hospitals.

Structured documentation frameworks have broader implications beyond compliance. Ray-Barruel et al. demonstrated that the I-DECIDED® tool improved not only record-keeping but also clinical outcomes, including reduced catheter-related complications [13]. Similarly, Hoskins et al. observed that structured observational studies continue to reveal persistent practice gaps, highlighting the need for ongoing audits and feedback [14]. Although our project did not directly measure patient outcomes, the improved documentation of complications in Cycle 2 indicates that such benefits may be anticipated with sustained use of the intervention.

From a health system perspective, improved documentation also strengthens medico-legal accountability and facilitates continuity of care. Reliable records enable better communication between shifts, departments, and specialties, reducing the risk of duplication or errors in cannula management. In resource-limited settings, where patient loads are high and staff turnover is frequent, structured documentation tools provide a safeguard against omissions that could otherwise compromise patient safety. Furthermore, comprehensive records create a valuable data source for future audits and infection prevention strategies.

This project has limitations. It was conducted in a single institution with a relatively small sample size, limiting generalizability. The relatively short follow-up period also does not establish whether improvements will be sustained long-term. In addition, internal validity may have been influenced by observer bias during data collection, potential Hawthorne effects whereby staff performance improved temporarily due to awareness of being observed, and the limited representativeness of the sample, which may not fully reflect broader hospital practices. Future audits should explore the durability of change, include multicenter data, and evaluate patient outcomes such as rates of phlebitis or catheter-related bloodstream infection. Despite these limitations, the magnitude of improvement observed across all parameters strongly suggests that the intervention had a genuine system-level impact.

In summary, this study demonstrates that substantial improvements in IV cannula documentation can be achieved through structured, low-cost interventions tailored to local realities. By embedding a standardized sticker into daily practice and reinforcing its use through staff education and ward-level reminders, compliance with documentation standards improved to near-universal levels. The approach is scalable, sustainable, and highly relevant to resource-limited hospitals. Future work should focus on improving maintenance documentation, assessing long-term sustainability, and extending this model to other invasive procedures.

## Conclusions

This QI project demonstrated that introducing a structured IV cannula documentation sticker, reinforced through staff training and ward-level reminders, led to dramatic improvements in compliance with documentation standards, achieving near-universal adherence across most parameters. The intervention proved effective, sustainable, and low-cost in a resource-limited setting, underscoring its value for enhancing patient safety, continuity of care, and accountability. Sustaining progress will require ongoing education, periodic re-auditing, and focused attention on maintenance documentation, with the potential for this model to be scaled to other invasive procedures.

## Figures and Tables

**Figure 1:**
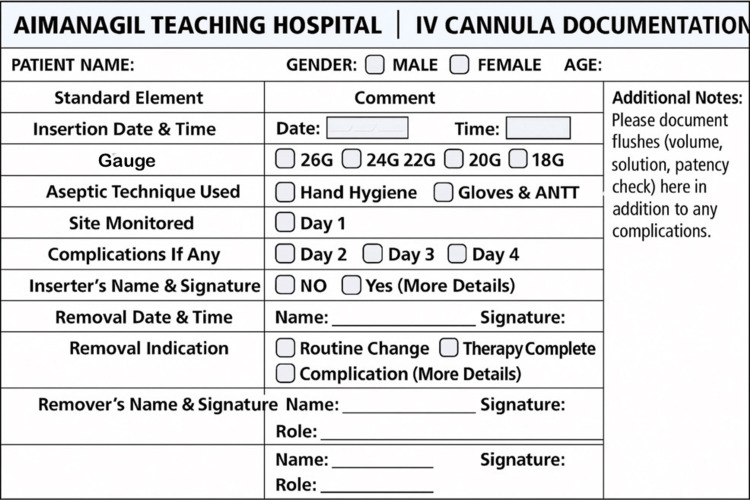
Intravenous cannula documentation sticker used at Al-Managil Teaching Hospital This standardized sticker was designed to ensure accurate and comprehensive documentation of intravenous (IV) cannula insertion and removal. It includes fields for patient demographics, cannula gauge and site, insertion indication, aseptic technique, site monitoring, complications, and the names/signatures of inserters and removers. The form also captures the date and time of both insertion and removal, as well as the reason for removal (routine change, therapy completion, or complication). An additional notes section allows staff to document flushes, solution type, patency checks, and any complications encountered. ANTT: aseptic non-touch technique

## Appendices

Appendix 1 
